# Hyperspectral imaging and artificial intelligence enhance remote phenotyping of grapevine rootstock influence on whole vine photosynthesis

**DOI:** 10.3389/fpls.2024.1409821

**Published:** 2024-09-19

**Authors:** Prakriti Sharma, Imasha Thilakarathna, Anne Fennell

**Affiliations:** Agronomy, Horticulture, and Plant Science, South Dakota State University, Brookings, SD, United States

**Keywords:** *V. hybrid* ‘Marquette’, graft, *Vitis*, daylength, convolutional neural network, computer vision, deep learning, transfer learning

## Abstract

Rootstocks are gaining importance in viticulture as a strategy to combat abiotic challenges, as well as enhancing scion physiology. Photosynthetic parameters such as maximum rate of carboxylation of RuBP (V_cmax_) and the maximum rate of electron transport driving RuBP regeneration (J_max_) have been identified as ideal targets for potential influence by rootstock and breeding. However, leaf specific direct measurement of these photosynthetic parameters is time consuming, limiting the information scope and the number of individuals that can be screened. This study aims to overcome these limitations by employing hyperspectral imaging combined with artificial intelligence (AI) to predict these key photosynthetic traits at the canopy level. Hyperspectral imaging captures detailed optical properties across a broad range of wavelengths (400 to 1000 nm), enabling use of all wavelengths in a comprehensive analysis of the entire vine’s photosynthetic performance (V_cmax_ and J_max_). Artificial intelligence-based prediction models that blend the strength of deep learning and machine learning were developed using two growing seasons data measured post-solstice at 15 h, 14 h, 13 h and 12 h daylengths for *Vitis hybrid* ‘Marquette’ grafted to five commercial rootstocks and ‘Marquette’ grafted to ‘Marquette’. Significant differences in photosynthetic efficiency (V_cmax_ and J_max_) were noted for both direct and indirect measurements for the six rootstocks, indicating that rootstock genotype and daylength have a significant influence on scion photosynthesis. Evaluation of multiple feature-extraction algorithms indicated the proposed *Vitis* base model incorporating a 1D-Convolutional neural Network (CNN) had the best prediction performance with a R^2^ of 0.60 for V_cmax_ and J_max_. Inclusion of weather and chlorophyll parameters slightly improved model performance for both photosynthetic parameters. Integrating AI with hyperspectral remote phenotyping provides potential for high-throughput whole vine assessment of photosynthetic performance and selection of rootstock genotypes that confer improved photosynthetic performance potential in the scion.

## 1 Introduction

Most of the commercial grapevines are grafted to improve their growth, physiology, and sustainability in diverse soil types. Many studies have indicated that it can lead to changes in source-sink relations, modifying carbon dynamics that can impact the overall performance of the vine (Di Filippo and Vila, 2011; Jones et al., 2009). Nevertheless, the literature on the physiological and molecular influence of rootstock mediated influence is very dependent on the rootstock and scion genotypes and environmental conditions of the studies.

Carbon gain is an outcome of plants leveraging photosynthesis to transform carbon dioxide (CO_2_) and water into organic compounds. Various studies have shown that the interaction between rootstock and scion has a significant impact on photosynthesis (Koundouras et al., 2008; Nimbolkar et al., 2016; Zhang et al., 2016). In grapevines, the rootstock is found to impact carbon gain through regulation of stomatal conductance in stress conditions (Shinozaki and Yamaguchi-Shinozaki, 2007). The rootstock is also found to impact photosynthetic rate by increasing carboxylation efficiency and net CO_2_ assimilation rate (Düring, 1994; Koundouras et al., 2008). Consequently, the underlying mechanisms through which rootstock affects photosynthesis remain ambiguous, necessitating research at both the molecular and physiological levels to enable the effective choice of rootstock for optimizing photosynthetic efficiency to enhance carbon gain in grapevine.

Selection of rootstocks for an improved conferred photosynthetic capacity phenotype is a complex and lengthy process (Cousins, 2005). Therefore, high-throughput measures are required to effectively select rootstocks that confer enhanced photosynthetic capacity in the scion. Direct measurement of photosynthesis using infrared gas analyzers (IRGA), can be employed to estimate light and CO_2_ curves that are used to gain photosynthetic mechanistic information (Farquhar et al., 1980; Von Caemmerer and Farquhar, 1981). Indeed, photosynthetic system derived biochemical kinetic metrics like 1) maximum rate of carboxylation of RuBP (V_cmax_) and 2) maximum rate of electron transport driving RuBP regeneration (J_max_) together with biochemical modeling is extensively used to understand photosynthetic performance in plants (Long and Bernacchi, 2003). However, direct gas exchange measurements require a long leaf acclimation time inside the measuring cuvette and accurate regulation of cuvette environment, which is best achieved under relatively constant ambient environments (Haworth et al., 2018). Thus, to maintain similar environments across measures, the gas-exchange measurements are typically performed for only a small portion of the day. Rapid A/Ci response curves (RACiR) plotting the relationship between net photosynthetic rate and CO_2_ concentration has been introduced, yet the technique is still lengthy and unsuitable for high-throughput of large sample numbers (Stinziano et al., 2019). In addition, grapevine photosynthesis is better understood as a characteristic at the vine canopy level, rather than at a single leaf level as found with IRGA measurements (Fu et al., 2022). Therefore, it is important to explore other remote or proximal sensing technologies that indirectly assess photosynthesis at the canopy level.

Hyperspectral imaging features from visible to near-infrared spectrum, for each pixel within the canopy area can be used as proxy for photosynthetic efficiency (Watt et al., 2020; Zarco-Tejada et al., 2016). Few studies have used hyperspectral information to estimate grapevine canopy level V_cmax_ and J_max_ parameters, highlighting the need for validation experiments to investigate the relationship between grapevine leaf and canopy level photosynthesis measurements (Asner et al., 2015; Barnes et al., 2017; Camino et al., 2022). As shown in aspen, cottonwood and other crops, hyperspectral reflectance measures may be integrated with several AI-based modelling approaches to predict ground based photosynthetic parameters (Fu et al., 2020; Serbin et al., 2012). Indeed, recent trends show a significant use of deep learning algorithms for the prediction of photosynthetic parameters using hyperspectral imagery (Furbank et al., 2021; Yu et al., 2022). However, a major challenge while implementing deep learning algorithms in physiological trait prediction is the limitation of ground-truth data samples. A hybrid model incorporating feature extraction using deep learning and classification/regression tasks with traditional ML algorithms is often employed to address this (Nguyen et al., 2021). The convolutional neural network (CNN) is extensively used for extracting spatial-spectral features, for predicting plant photosynthetic pigments and parameters (Deng et al., 2024; Prilianti et al., 2021; Zhang et al., 2022). Use of 1D-CNN and 2D-CNN for extraction of patterns in the hyperspectral signal have shown a higher prediction accuracy of photosynthetic parameters than traditional machine learning algorithms (Prilianti et al., 2019; Prilianti et al., 2021; Zhang et al., 2022). Another approach to address the issue of limited ground-truth data samples, is to use transfer learning algorithms as a feature extractor (Tan et al., 2018; Weiss et al., 2016). This method uses a pre-trained model to transform unprocessed data into a collection of features that can be comprehended by a machine learning model to extract pertinent patterns or characteristics. These machine learning models demonstrate superior generalization across networks and rapid convergence speeds (Alzubaidi et al., 2021).

The challenge of effectively extracting and utilizing both spectral and spatial information found in hyperspectral data persists. Our study addresses this challenge through a proposed hybrid model that blends the strength of both deep learning and machine learning techniques to unlock the potential of dimensional hyperspectral data for photosynthesis prediction. The hybrid approach used here is based on powerful feature extraction algorithms to extract significant information from the hyperspectral data for one- and three-dimensional datasets. This study leverages models like principal component analysis (PCA) (Wold et al., 1987), autoencoders (Bank et al., 2023) and 1D-CNN as feature extraction algorithm for 1-D hyperspectral data. For 3-D hyperspectral data, the approach incorporates several transfer learning algorithms such as VGG16 (a widely used feature extractor in computer vision applications (Pardede et al., 2021; Simonyan and Zisserman, 2014; Tammina, 2019)) and Inception-ResNet (which combines inception modules and residual connections (Ferreira et al., 2018)) and two-dimensional convolutional neural networks (2D-CNN). In addition to performing feature extraction, the hybrid model integrates XGBoost (eXtreme Gradient Boosting) (Chen and Guestrin, 2016) to tackle regression tasks, leveraging decision trees as base learners in a boosting technique where models are sequentially added until the error is minimized (Chen and Guestrin, 2016).

The scope of this research is to assess the influence of rootstocks on scion photosynthetic parameters and determine the accuracy of hyperspectral imagery in predicting these phenomena. Accordingly, the study sets forth the following specific objectives: 1) to conduct a comparative study of photosynthetic efficiency in various rootstocks using both direct (ground-truth) and indirect (spectral) methods; 2) to confirm the effectiveness of hyperspectral remote sensing in accurately measuring photosynthesis by incorporating AI-based algorithms for feature extraction of hyperspectral data. This study uses a ground based remote phenotyping hyperspectral system to capture the vines’ extensive vertically distributed canopy rather than an aerial system that only captures the smaller top portion of the grapevine canopy. Further, the measurements were made throughout the season to capture photosynthesis during the natural declining daylength. Evaluation of the hybrid model here uses all wavelengths measured instead of specific wavelengths that have been related to photosynthetic parameters in other remote sensing studies.

## 2 Methods

### 2.1 Plant materials

*Vitis* hybrid ‘Marquette’ grafted to five commercial rootstocks 1103 Paulsen (1103P), 3309 Couderc (3309 C), Teleki 5C (5C), Freedom (FREE), Selection Oppenheim 4 (SO4), and ‘Marquette’ grafted to ‘Marquette’ (homograft) (**Table 1**) were used to measure rootstock influence on photosynthetic parameters. Vines were custom grafted in 2018, using same aged dormant cane materials, by Double A Vineyards (Fredonia, NY, USA) and grown for one year prior to planting. The Marquette homograft provides the inherent photosynthetic characteristics of the ‘Marquette’ genotype in a grafted vine so that the conferred influence from the commercial rootstocks on the Marquette scion can be determined in direct comparison of grafted vines. There were four replicates for each graft combination, organized in four complete blocks that were randomly placed within four rows (1 block per row) of a larger experimental vineyard.

**Table 1:** Rootstock genotypes pedigree and characteristics.

Rootstock	Pedigree	Characteristics
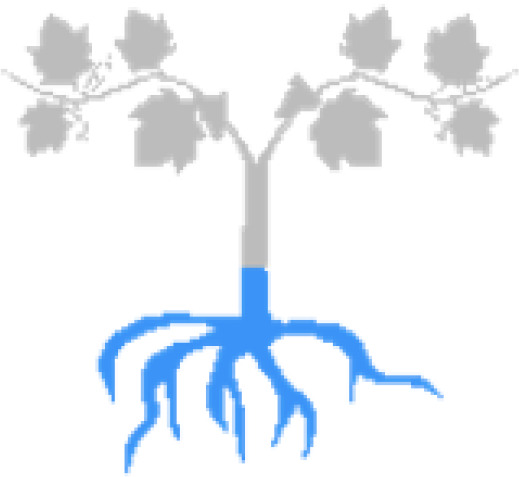 *1103 Paulsen*	*V. berlandieri* Planch. and *V. rupestris* Scheele	- expanded, deep branching roots, high resistance to phylloxera, adapted to a wide range of soil conditions - good grafting aptitude, confers a high vigor, long vegetative cycle and delays ripening.
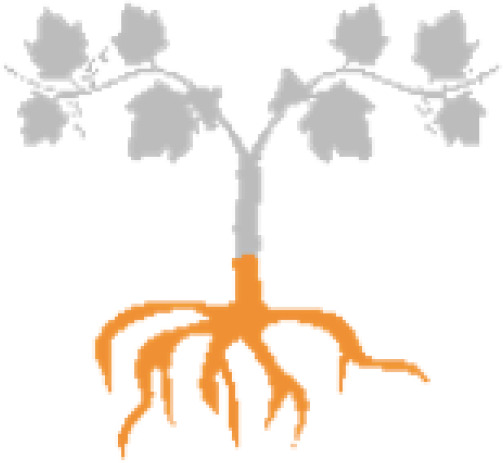 *3309 Couderc*	*V. riparia* Michx. and *V. rupestris* Scheele ‘Martin’	- slow root system generation, expanded root types, sensitive to water stress and Mg, N, B and K, good resistance to phylloxera - Good affinity to grafts, confer low to moderate vigor
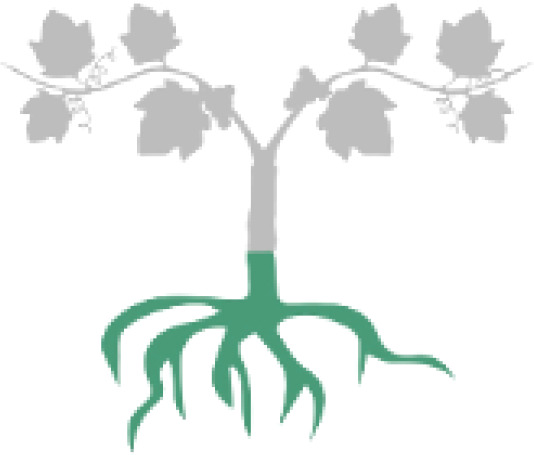 *Teleki 5C (5C)*	*V. berlandieri Planch.* and *V. riparia* Michx.	- Low to medium tolerance to different soil conditions (drought, salinity, lime), high resistance to phylloxera. - tends to have a low yield-to-pruning ratio and is well suited for varieties with poor fruit set.
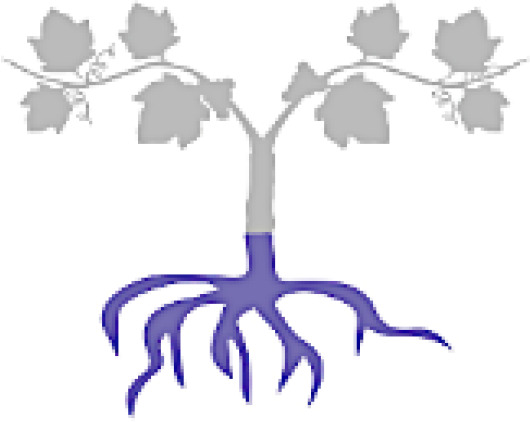 *Freedom*	*1613 (V. Longii Prince* ‘solonis’ *x V. hybrid* ‘Othello’*)* and *V. champini*	- Low to medium tolerance to different soil conditions (drought, salinity, lime) - Susceptible to phylloxera but resistant to broad spectrum of nematodes - Confers high vigor, sensitive to latent viruses
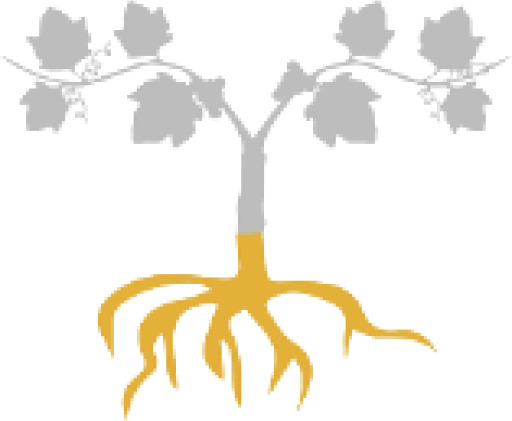 *Selection Oppenheim 4 (SO4)*	*V. berlandieri* Planch. *and V. riparia* Michx.	- Highly tolerant to phylloxera, moderate vigor rootstock (low vigor in first years of development), compatible with grafts but limited radial trunk growth - impacts on early development and maturity of scion
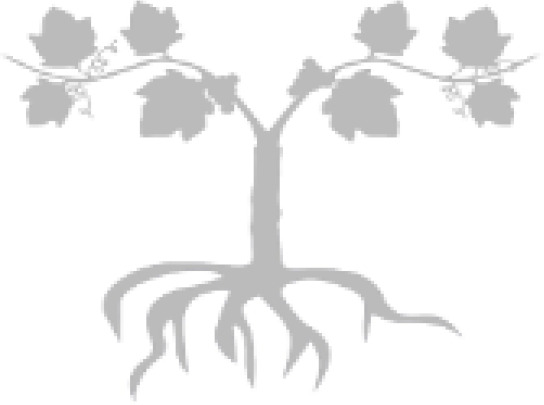 *Marquette*	*V. hybrid (MN 1094 and Ravat 262)*	- Cold hardy resistant - Resistant to common grape diseases (downy and powdery mildew) and moderate resistant to foliar phylloxera - Used as control in this study

Vine photosynthetic and hyperspectral profiles were measured in the field at the South Dakota State University research vineyard in Brookings, SD (44.3114 °N, -96.7984 °W). A high cordon management system was imposed on vines in 2020. The vines had a 1.828 m spacing within the row and 3.048 m between rows. Vines were maintained with fruit during measurement years (2022 and 2023). The vineyard used an automated irrigation system to supplement natural precipitation when less than 5.08 cm/month from the flowering stage until grape maturation. Both direct photosynthetic measurements and hyperspectral profiling was carried out post-summer solstice in 2022 and 2023, targeting daylengths of 15 h, 14 h, 13 h, and 12 h. These time points were chosen as summer solstice, the longest day of year (June 20, 15 h 31 min), occurs about 2 weeks after flowering and subsequently daylength begins decreasing, the sampling period was chosen to cover photosynthetic activity during fruit development and ripening period. Measurements were taken between 9:00 am and 12:00 pm to minimize the potential influence of large environmental fluctuations.

### 2.2 Direct measurement of photosynthesis attributes using infrared gas analyzer

The ground-truth data was acquired using a LI-COR (Li-6800, LICOR Biosciences, Lincoln, NE, USA) portable photosynthesis system. The LI-COR settings were fixed for the temporal measurements: Flow rate of 600 μmols^-1^, temperature and relative humidity set closest to ambient conditions, reference CO_2_ to 400 μmol mol^-1^, and saturating light of 1800 μmolm^-2^ s^-1^. One leaf from mid-shoot for each vine was chosen for measurement, providing four replicate samples per genotype (six genotypes), totaling 24 samples (6 genotypes x 4 replicates) for each sampling date. The selected leaves were fully developed, healthy middle leaves, adapted to sunlight conditions to ensure uniformity in photosynthetic measurements and minimize potential variability due to leaf development stages or environmental factors. The Rapid A/Ci curves (RACiR, net CO_2_ assimilation rate A, versus calculated substomatal CO_2_ concentration, Ci) was measured to derive photosynthetic parameters from a clamped leaf area of 6 cm².The Farquhar-Berry-von Caemmerer model was used to fit A/Ci curve to derive photosynthetic capacity in the vines in terms of maximum rate of carboxylation of RuBP (V_cmax_) and maximum rate of electron transport driving RuBP regeneration (J_max_) (Farquhar et al., 1980). The R package ‘racir’ (Stinziano et al., 2019) was used to perform calibration fits which allowed to select appropriate polynomial fit based on AIC criterion. Selected fit was used to derive V_cmax_ and J_max_ using package in R ‘plantecophys’ (Duursma, 2015).

### 2.3 Indirect measurement of photosynthesis using hyperspectral remote sensing

The hyperspectral sensor SPECIM IQ (Specim, Spectral Imaging Ltd., Oulu, Finland) was used to collect canopy reflectance measure to predict photosynthetic parameters V_cmax_ and J_max_ (Behmann et al., 2018; Deng et al., 2024). The operation hardware of the Specim IQ sensor utilizes push broom technology where it simultaneously captures a single spatial line of the image with the entire wavelength spectrum, then moves to the next line. It acquires reflectance for 204 narrow wavelength bands with a spectral range of 397nm to 1000nm with a spectral resolution of 7nm. This sensor acquires spectral information in line scanning of 512 pixels, resulting in static image size of 512 by 512 pixels. The viewing area is 0.55 m by 0.55 m., which achieves a spatial resolution of 1.07 mm when placed at one meter from the object. For this study, the hyperspectral sensor was placed one meter from the trellis wire used for the vines. A Spectralon white reference panel (Specim, Spectral Imaging Ltd., Oulu, Finland) was placed next to the vine to ensure calibration of radiance image. The sensor built-in function was used for digital number to reflectance conversion. Hyperspectral data was collected using the default recording option for saving hyperspectral data cubes, to generate the unprocessed reflectance data. The images were processed using ENVI software (L3Harris Geospatial Solutions Inc., Broomfield, CO, USA). First, the canopy surfaces consisting only of leaves, were extracted as the region of interest (ROI) and background pixels were omitted. The radial basis kernel function in support vector machine (SVM) classifier was used to create binary layer that eliminated shadow and background pixels from the imagery. The accuracy of SVM in this process was found to range from 96 to 98.12%. The end bands were eliminated thereby reducing background noise and resulted 187 total wavelengths for further analysis. Images were subsequently resized to a 250x250 dimension to decrease the computational time required for the modeling algorithms. Similarly, one-dimensional spectral data were extracted from the leaf ROI (Region of Interest) areas, which were used as input features in some models.

### 2.4 Environmental and chlorophyll features

Additional meteorological parameters and leaf chlorophyll values were measured for use in modeling photosynthetic parameters in combination with the hyperspectral features. Chlorophyll levels were measured at the same time as the LI-6800 photosynthesis measurements using the MC-100 Chlorophyll Concentration Meter (Apogee Instruments, Inc., UT, USA) for the ground-truth data collection (Parry et al., 2014). For these measurements, the instrument was configured to the ‘GRAPE’ option in its selection menu. Three fully grown middle leaves were sampled from each genotype, and the average value was calculated. The temperature, solar radiation (SR), and relative humidity (RH) were recorded by SDmesonet station situated at the vineyard site in South Dakota State University (SDmesonet, 2023). The real time weather data corresponding to the time when CO_2_ assimilation curves were measured for each genotype were retrieved for each sampling date. The mean of temperature, solar radiation (SR), and relative humidity (RH) were determined by averaging the maximum and minimum values recorded during the specified sampling hour. These averages were used as model input parameters during the second phase of model analysis.

### 2.5 Statistical analysis

#### 2.5.1 Data exploration of direct and indirect photosynthetic measures

Ground-truth photosynthetic data was normalized by z-score transformation and then a two-way ANOVA was performed to determine whether there were significant differences between genotypes and measurements taken at specified daylengths (15, 14, 13, and 12 h) (Abdi and Williams, 2010). The ‘ggplot’ package in R was employed to create visual representations, using violin plots to demonstrate the genotypic differences across both years, as well as line plots to depict the trend of photosynthesis in relation to changes in daylength (Wickham et al., 2016). A principal component analysis was conducted to determine genotypic differences based on their spectral signature using built-in ‘stats’ package in R (Team et al., 2018). This method was also applied to determine if spectral signature varied based on environmental settings.

#### 2.5.2 Prediction model development

For the model development, the focus was solely on development of integrated/hybrid model. To predict V_cmax_ and J_max_ independently, both one-dimensional hyperspectral data and three-dimensional data in image format were used. Multiple of algorithms were employed to extract features, these were combined with the XGBoost algorithm for prediction purposes, resulting in the formation of an advanced hybrid model (Chen and Guestrin, 2016). For one-dimensional hyperspectral data (spectral), the algorithms included principal component analysis (PCA), autoencoders, and proposed one-dimensional convolutional neural network (VIT-CNN1D) (Wold et al., 1987). For three-dimensional hyperspectral data (spectral-spatial), different transfer learning techniques such as VGG16, Inception-ResNet, and a proposed two-dimensional convolutional neural network model (VIT-CNN2D) was applied (Simonyan and Zisserman, 2014).

For model assessment, the complete dataset of both years measured was split into two parts: 80% for training and 20% for testing. During the training of all the models, a resampling strategy employing 10-fold cross-validation was implemented (Fushiki, 2011). Additionally, hyperparameter optimization was conducted to select the most suitable parameters for each model. To evaluate the performance of the models, three important evaluation metrics were used to analyze both the training and testing predictions. These metrics were Root Mean Square Error (RMSE), Mean Absolute Error (MAE), and the Coefficient of Determination (R²).


$$\;RMSE=\sqrt{\frac{1}{n}\sum_{i=1}^{n}{\left(\;y_{i}\;–\;\hat{y_{i\;}}\right)}^{2}}$$

$$MAE=\frac{1}{n}\sum_{i=1}^{n}|y_{i}\;–\;\hat{y_{i\;}}|\;$$

$$R^{2}=1-\;\frac{\sum_{i}{\left(\;y_{i}\;–\;\hat{y_{i\;}}\;\right)}^{2}}{\sum_{i\;}\left(\;y_{i}-\;\frac{1}{n}\;\sum_{i=1}^{n}y_{i\;}\right)}\;$$

### 2.6 Model feature extraction and prediction assignment algorithms

#### 2.6.1 Principal component analysis

Fifteen PC components, that captured more than 99% of total variation of spectral features were selected for this study to predict photosynthetic parameters. PCA implementation was done in Python using the scikit-learn library (Pedregosa et al., 2011).

#### 2.6.2 Autoencoders

The autoencoder models used in this study were built using a set of hyperparameters, allowing for the broad assessment of various architectural configurations. Two dense layers with the activation function Rectified Linear Input (ReLU) were designed for encoders (Agarap, 2018). Hyperparameters were used to specify the units for these layers. For the decoder, a single dense layer followed by the final output layer, units were set at 50. The parameters for the model compilation were the mean squared error (MSE) as the loss function and Adam as the optimizer (Kingma and Ba, 2014). Similarly, the ‘kerastuner’ package from TensorFlow was used to optimize an autoencoder model’s hyperparameters (**Table 2**) (Abadi et al., 2016).

**Table 2:** Hyperparameters and specifications for each model selection using random search cross validation.

Models	Hyperparameters	Specifications
**Autoencoders**	units	min:32, max:256, step = 32
**VIT-CNN1D**	Filters	min:16, max:128, step = 16
	kernel size	min:2, max:7
	Learning rate	min: 1e-4, max:1e-1
	Optimizer	Adam
	epoch	100 per trial
	loss function	Mean squared error
**VIT-CNN2D**	Filters	min:32, max:256, step = 16
	kernel size	min:2, max:7
	Learning rate	min: 1e-4, max:1e-1
	Optimizer	Adam
	epoch	100 per trial
	loss function	Mean squared error
**XGBoost**	maxdepth	min:2, max:10, step = 2
	Learning rate	min:0.01, max:0.1
	n_estimators	min:100, max:400
	min_child_weight	min:1, max:4
	reg_alpha	min:0, max:0.01

#### 2.6.3 VIT-CNN1D model

**Figure 1:**
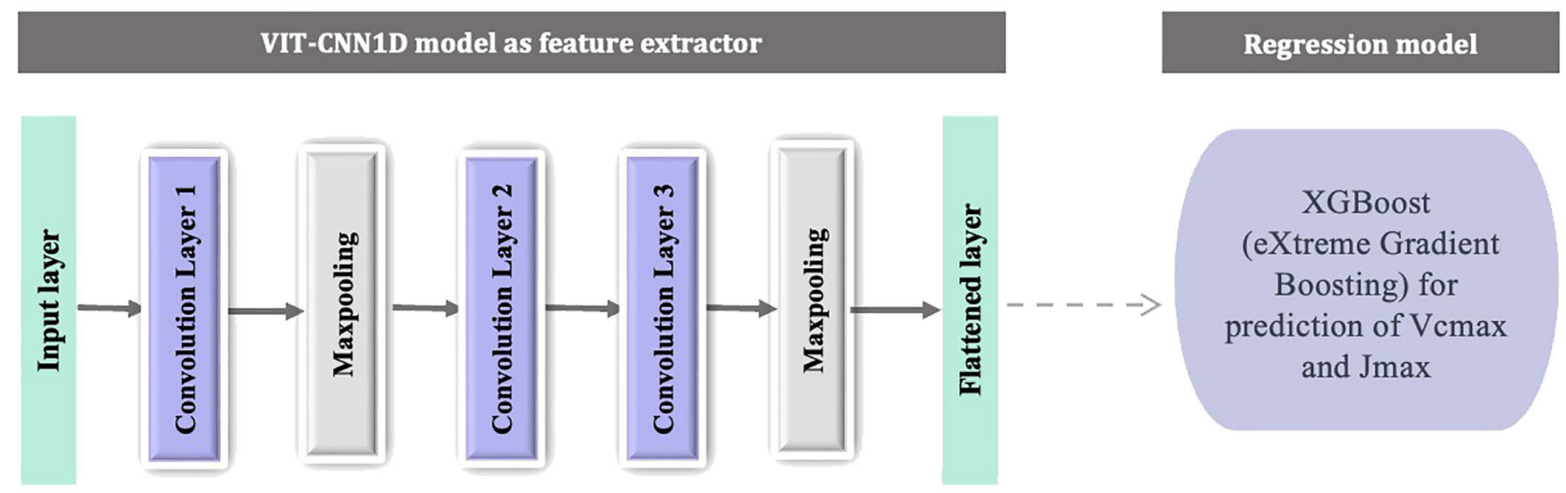
Schematic representation of two-stage machine learning model with components: a feature extraction module VIT-CNN1D (consists of three convolution layers and two pooling layers) and a regression model as XGBoost to predict V_cmax_ and J_max_.

The 1D-CNN models were implemented to derive pattern(s) or information across the spectral dimensions of hyperspectral data for prediction of photosynthetic parameters. This algorithm is defined as VIT-CNN1D, which was specifically designed for feature extraction from one-dimensional hyperspectral data. To meet the modeling goals of this research, the 1D-CNN was employed with hyperparameter optimization, utilizing the Tensorflow modules ‘kerastuner’ (Abadi et al., 2016; Pon and KK, 2021). The CNN framework consisted of three convolutional layers and two pooling layers as shown in **Figure 1**. The Rectified Linear Input (ReLU) was employed as the convolutional output activation function (Agarap, 2018). The flattened layer was then connected to the output layer, derived with linear function. The hyper parameters i.e., filter size, kernel size and learning rate were selected using *RandomSearch* that operates in hyperparameter combinations at random in attempt to discover the best effective set for a given model (**Table 2**) (Bergstra and Bengio, 2012; Li and Talwalkar, 2020). Using 15 trials, the model with the smallest validation mean squared error was selected and flattened layer were extracted as features. The resultant spectral features were then passed on as input for prediction task for estimating photosynthetic parameters.

#### 2.6.4 Transfer learning approaches (VGG16, Inception-ResNet)

The VGG16 architecture was used only as a feature extraction technique, such that top layers were omitted and pre-trained weights were excluded, to implement the model from scratch on our dataset (Simonyan and Zisserman, 2014). The input dimensions of 250*250*187 were used which indicated that the model was modified to process data with more channels. The custom VGG16 configuration was used to retrieve features from original images, which were then used to feed XGBoost algorithm for the regression task (Chen and Guestrin, 2016). Similarly, the InceptionResNetV2 model (Inception-ResNet) was altered to specifically operate for this study by excluding top classification layers and pretrained weights (Szegedy et al., 2017).

#### 2.6.5 VIT-CNN2D

**Figure 2:**
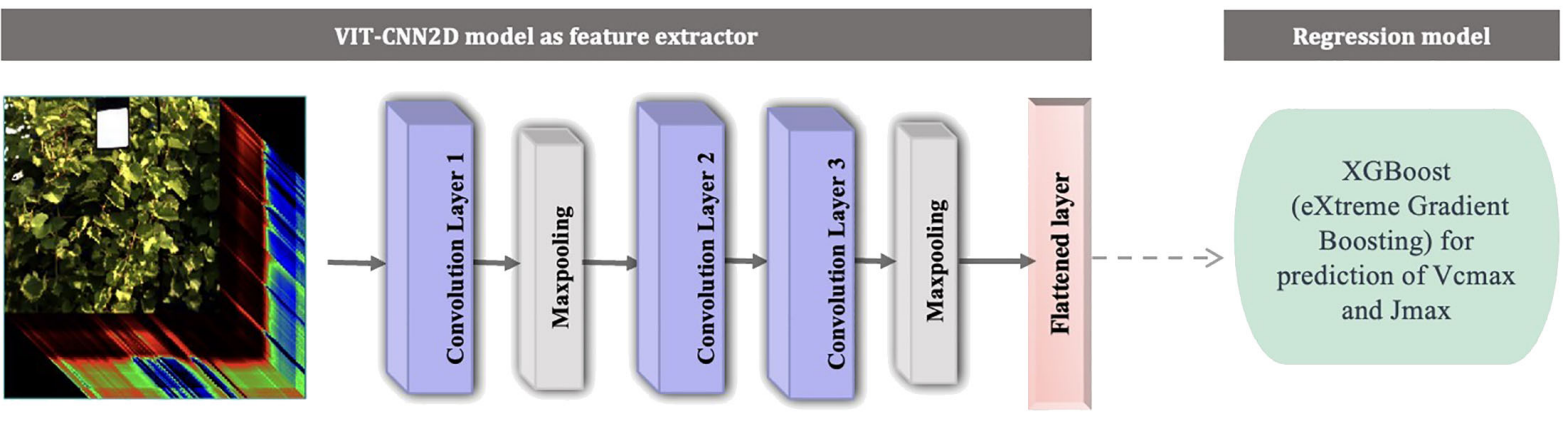
Schematic representation of two-stage machine learning model with components: a feature extraction module as VIT-CNN2D (consists of three convolution layers and two pooling layers) and a regression model as XGBoost to predict V_cmax_ and J_max_.

The VIT-CNN2D architecture defined in this study was used for extracting features from hyperspectral data cube. Since the ground-truth data size was very small, the architecture was kept fairly simple, like the VIT-CNN1D with 3 convolutional layers and 2 pooling layers as shown in **Figure 2**. Similar to the previous scenarios, the Rectified Linear Input (ReLU) was used as the activation function for the convolutional output (Agarap, 2018). The hyperparameters, such as filter size, kernel size, and learning rate, were determined using *RandomSearch* (**Table 2**) (Bergstra and Bengio, 2012; Li and Talwalkar, 2020).

#### 2.6.6 XGBoost

After the hyperspectral feature extraction, XGBoost was used in this study for the regression task incorporating regularization to prevent overfitting (Chen and Guestrin, 2016). Model training was performed optimizing the hyperparameters such as learning rate, tree depth, number of trees used in model and regularization terms as described in **Table 2** using *RandomSearch* (Bergstra and Bengio, 2012). The scikit-learn library was used to implement the model workflow, consisting of 10-fold cross validation technique to ensure the robustness and generalizability of the trained model (Pedregosa et al., 2011). These parameters were used to prevent overfitting and assess the true predictive performance of the model before applying it to the test data.

### 2.7 Analysis with environmental data

Following the identification of the top-performing hybrid model during the initial phase analysis, chlorophyll concentration (CC) and weather parameters (temperature, SR, and RH) were incorporated to test their ability to improve model performance. To predict V_cmax_ and J_max_, the model was tested in two ways: first by incorporating chlorophyll values with the spectral data, and second by incorporating weather variables with the spectral data.

## 3 Results

### 3.1 Ground-truth measures for main factors of rootstocks genotype and daylength

**Figure 3:**
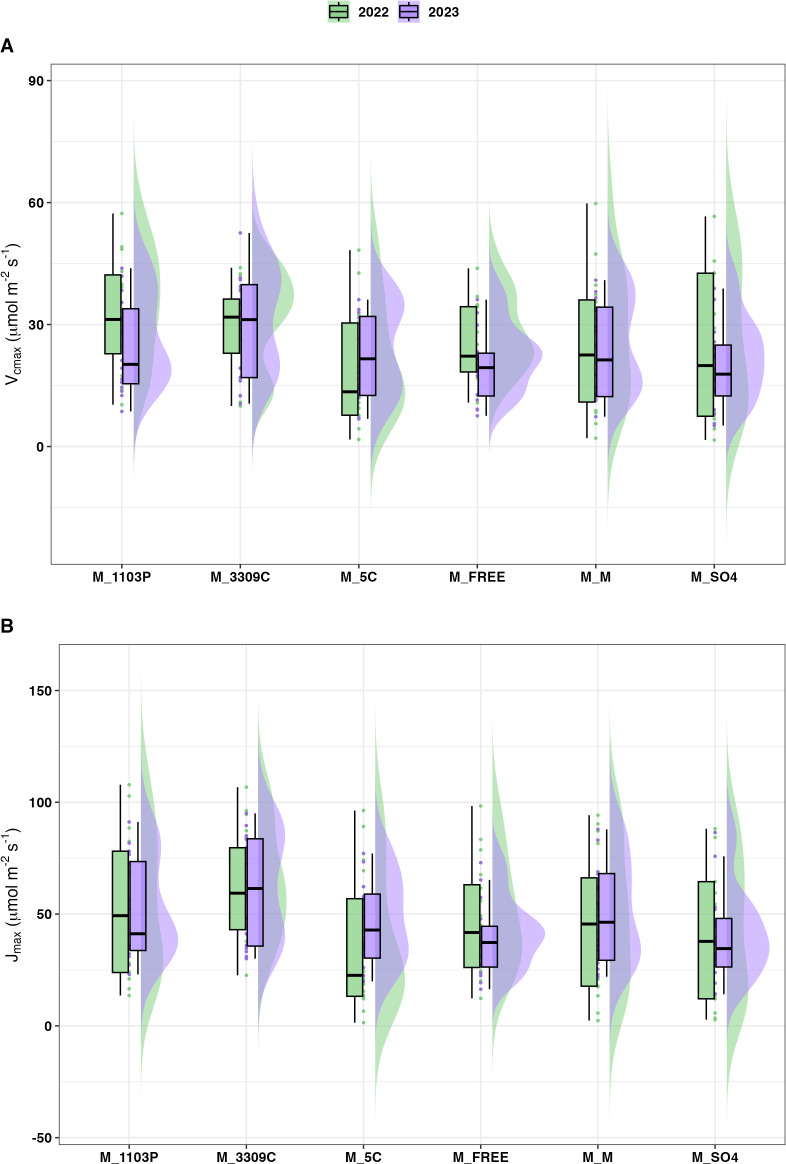
Distribution of V_cmax_ **(A)** and J_max_ **(B)** for all rootstocks in field conditions. The measures for each genotype are the cumulative measures for four different replicates of each graft combination sampled over different daylength conditions. Year of measure 2022 (green) and 2023 (purple), *V. hybrid* ‘Marquette’ common scion heterografted to rootstock 1103P, 3309C, 5C, Freedom (FREE), SO4 and homografted to ‘Marquette’.

The distribution pattern of V_cmax_ and J_max_ varied between grafted rootstock genotypes for 2022 and 2023 (**Figures 3A, B**). ‘Marquette’ on 3309C showed higher values compared to other combinations, including the homograft of ‘Marquette’ for both photosynthetic parameters. The main effects of genotype and daylength on V_cmax_ were significant and indicated the scion maximum rate of RuBP carboxylation differed significantly as influenced by rootstock genotypes (**Table 3**). ***Post-hoc*** analysis revealed that ‘Marquette’ on 3309C rootstock had the greatest average value for V_cmax_. ‘Marquette’ on 5C and SO4 had the lowest V_cmax_ and differed considerably from the other graft combinations. A similar trend was observed for J_max_, both the main effects for rootstock genotype and daylength were significant and the greatest average was observed for ‘Marquette’ on 3309C and 1103P, while the lowest average was observed for ‘Marquette’ on 5C.

**Table 3:** Photosynthetic parameter ANOVA.

A. Rootstock Genotype						
	M_1103P	M_3309C	M_5C	M_Freedom	M_SO4	M_Marquette
V_cmax_	0.31ab	0.35a	-0.32c	-0.16bc	-0.19c	-0.02abc
J_max_	0.17ab	0.51a	-0.28bc	-0.16bc	-0.28c	0.0bc
B. Daylength						
	**15 h**	**14 h**	**13 h**	**12 h**	
V_cmax_	0.73a	0.63a	-0.34b	-1.02c	
J_max_	0.91a	0.51b	-0.46c	-0.96d	

Initial measurements of V_cmax_ and J_max_ were recorded in molm^-2^s^-1^ prior to undergoing z-transformation for normalization conformity. A) Rootstock comparisons, B. Daylength comparisons. Within each row, mean values with common letters indicate no significant variance amongst them, as determined through Tukey’s Honest Significant Difference (HSD) test for multiple comparisons, n=4. M = V. hybrid ‘Marquette’ common scion heterografted to rootstock 1103P, 3309C, 5C, Freedom, SO4 and homografted to ‘Marquette’.

**Figure 4:**
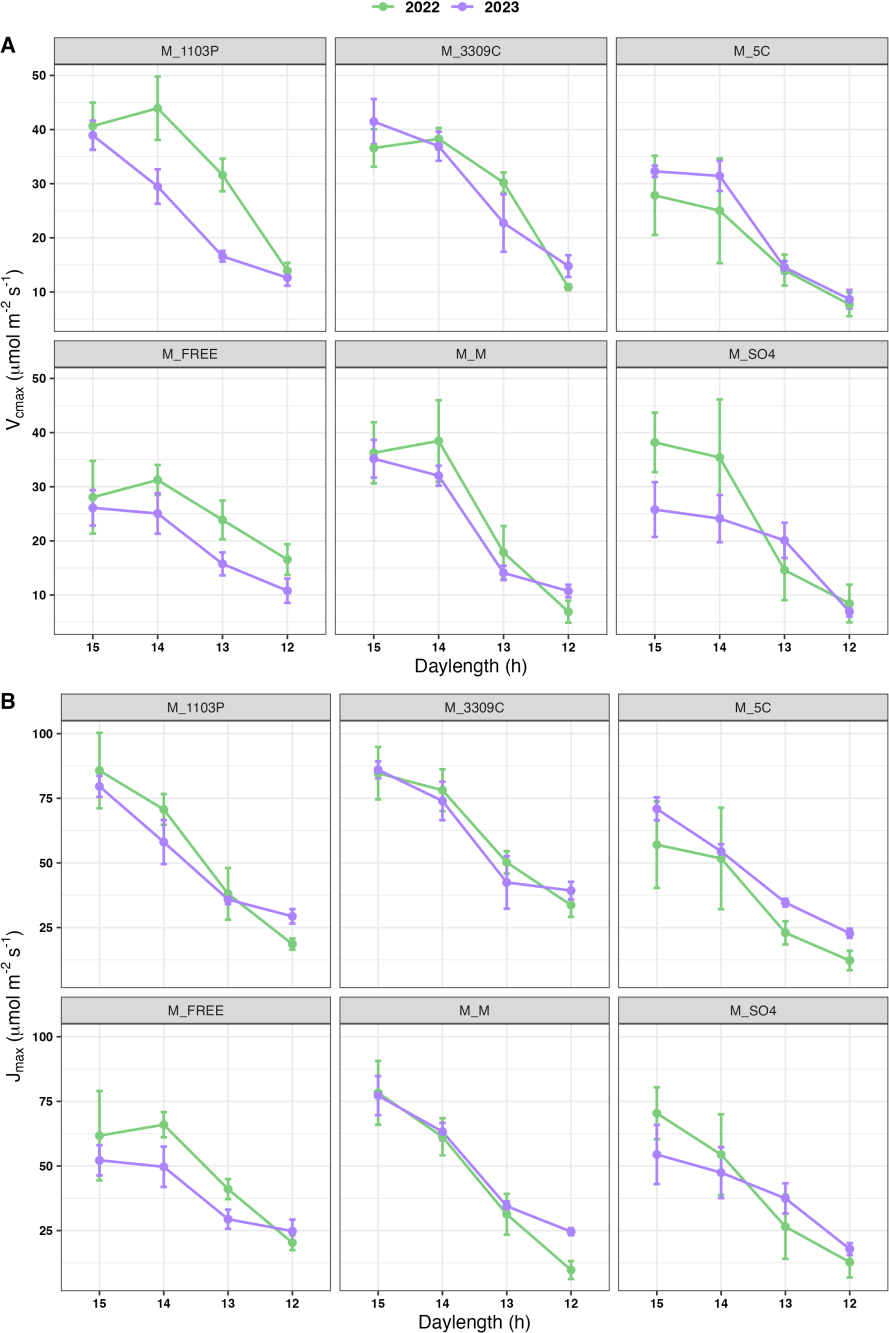
Temporal trend of V_cmax_ **(A)** and J_max_ **(B)** across the decreasing daylength in field conditions The measures for each rootstock combination in year 2022 (green) and 2023 (purple); mean values calculated for each daylength interval with standard error of the mean; M = V. hybrid ‘Marquette’ common scion heterografted to rootstock 1103P, 3309C, 5C, Freedom, SO4 and homografted to ‘Marquette’.

A decrease in V_cmax_ was detected for ‘Marquette’ on all genotypes including the homograft as the daylength hours progressed from 15 h to 12 h (**Figure 4A**). Similarly, a decrease in J_max_ was observed with daylength hour progression (**Figure 4B**). ***Post-hoc*** analysis also revealed that significant differences were observed across the different daylengths (**Table 3**).

### 3.2 Rootstock genotype induced hyperspectral response differences

**Figure 5:**
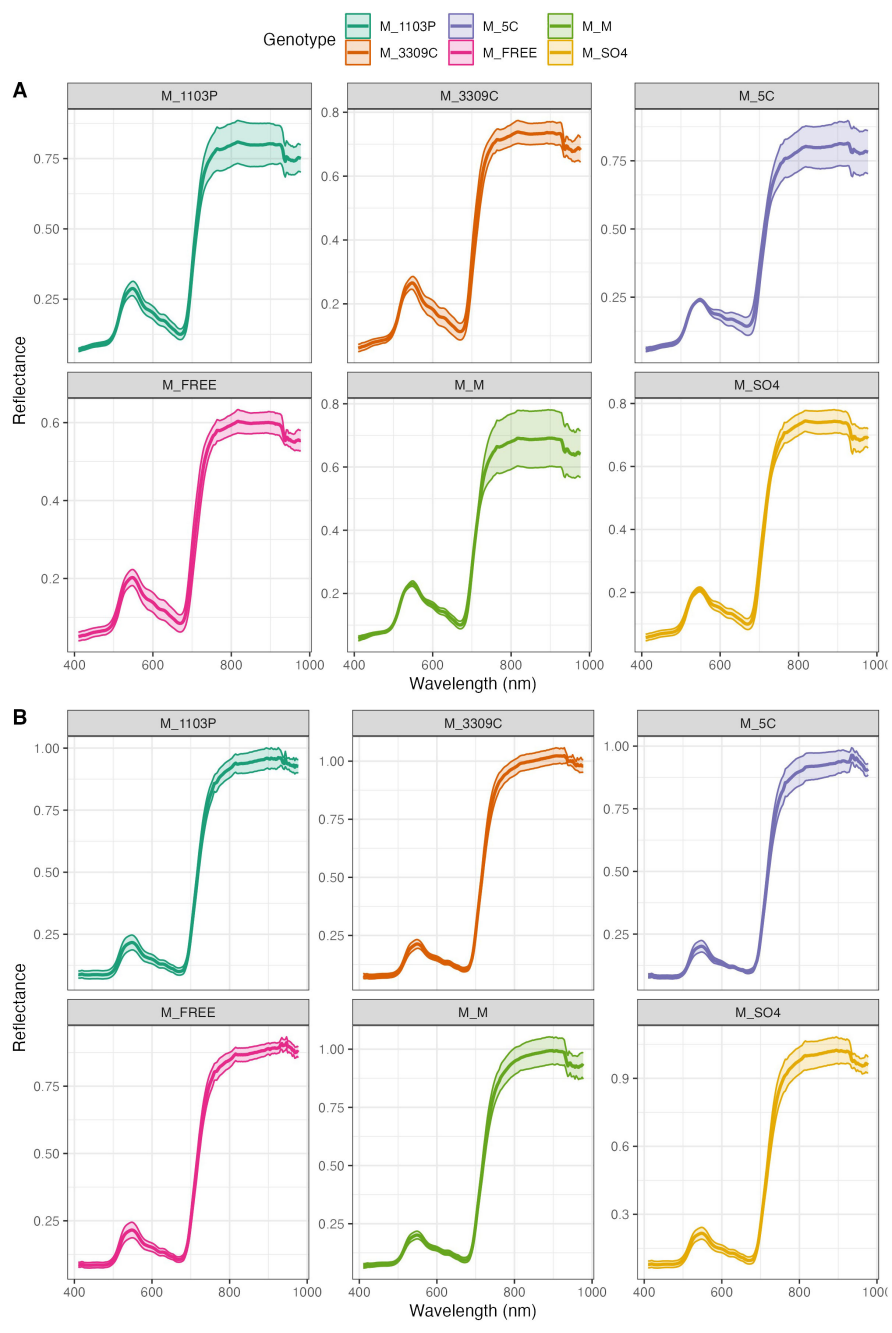
Average spectral signature for each rootstock combination in field at different daylengths. The hyperspectral reflectance derived for each rootstock combination in 2022 **(A)** and 2023 **(B)**; M = *V. hybrid* ‘Marquette’ common scion heterografted to rootstock 1103P, 3309C, 5C, Freedom, SO4 and homografted to ‘Marquette’.

**Figure 6:**
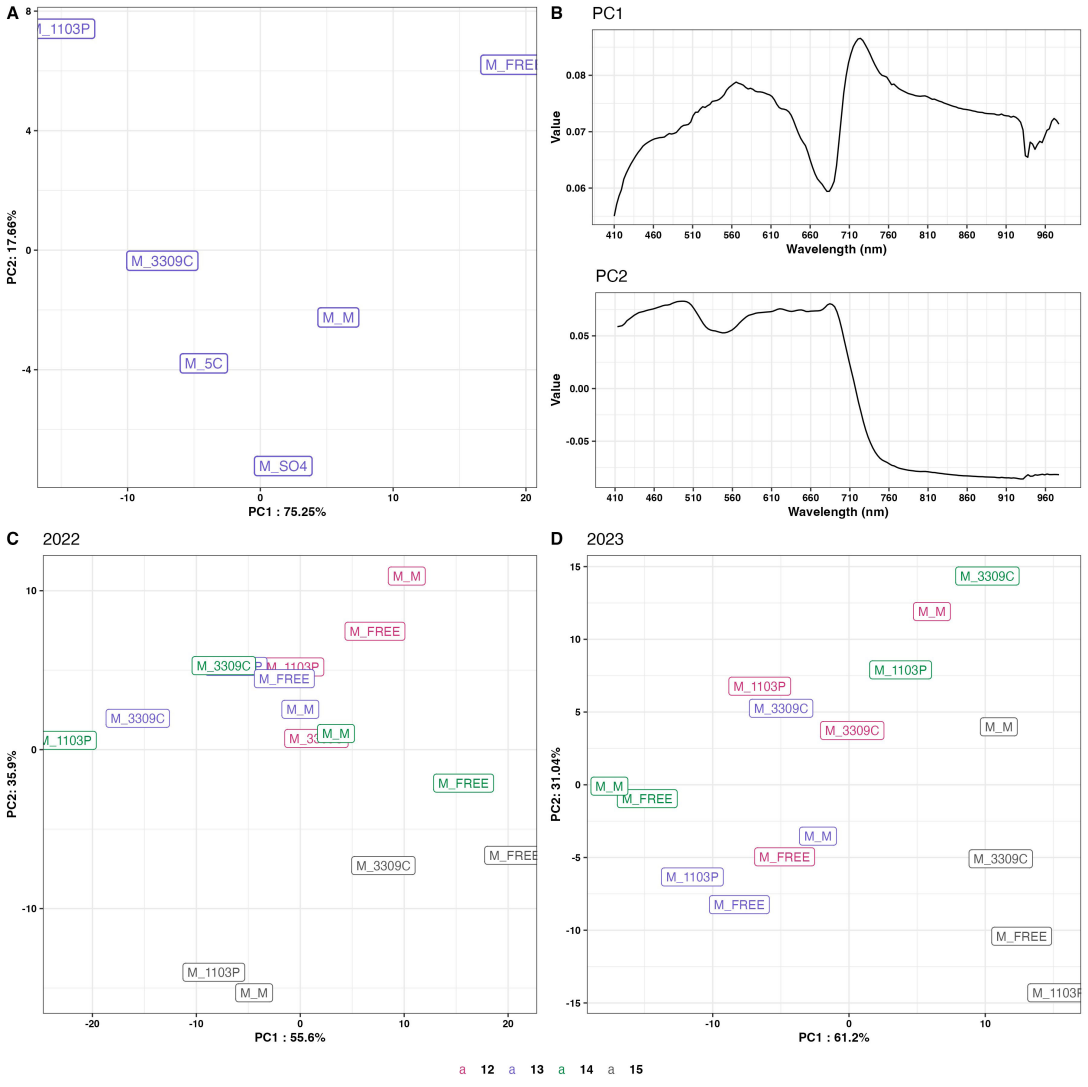
Principal component of spectral signature response as influenced by rootstock. **(A)** Data from all daylengths in 2022 and 2023; **(B)** PCA loadings from PC1 and PC2 derived from PCA in panel A; **(C, D)** PCA of 2022 and 2023 grafted rootstock response for all daylengths, 15h (black), 14 h (green), 13 h (blue), 12 h (pink). M = *V. hybrid* ‘Marquette’ common scion heterografted to rootstock 1103P, 3309C, 5C, Freedom, and SO4 and homografted to ‘Marquette’.

The genotypes showed different spectral signatures in response to a declining daylength (**Figure 5**). The genotype response is identified as reflectance on the y-axis in relation to the spectral bands measured on the x-axis. The variability around the central tendency is observed as the shaded region around the mean response for each wavelength (solid line) band. The greater the variability around the mean suggested that the response varied more across replicates or daylength conditions. The red-edge region, which is typically associated with chlorophyll absorption, is characterized by a substantial increase in reflectance that starts at approximately 700 nm across all genotypes. The spectra exhibit typical plant reflectance patterns, with peaks and troughs that correspond to specific absorption features, and their structure is consistent across genotypes. Nevertheless, there was genotype variation in wavelength reflectance patterns observed in 2022 (**Figure 5A**) and 2023 (**Figure 5B**). Also, there was a greater variation range for 2022 than in 2023. Vines of ‘Marquette’ grafted to 5C had wider regions (greater variation) than the other rootstocks for both years. The PCA (**Figure 6A**) revealed first principal component (PC1), explaining 75.25% of the variance in the data, while the second principal component (PC2), explaining 17.66% of the variance. The PCA space showed that the spectral characteristics of genotypes like ‘Marquette’ on 1103P and ‘Marquette’ on FREE were significantly different from those of other genotypes. On further analysis, variation associated with the first two principal components was primarily attributable to fluctuations in the 580 nm and 710 nm regions as shown by loading plots (**Figure 6B**). The variations in the spectral signature (**Figures 6C, D**) were significantly influenced by daylength. In both years, data for 15 h was in the lower right quartile of the PCA plot separated from the other daylengths (**Figures 6C, D**).

### 3.3 Prediction of photosynthetic parameters

#### 3.3.1 Prediction of V_cmax_ and J_max_ using different feature extraction algorithms

**Figure 7:**
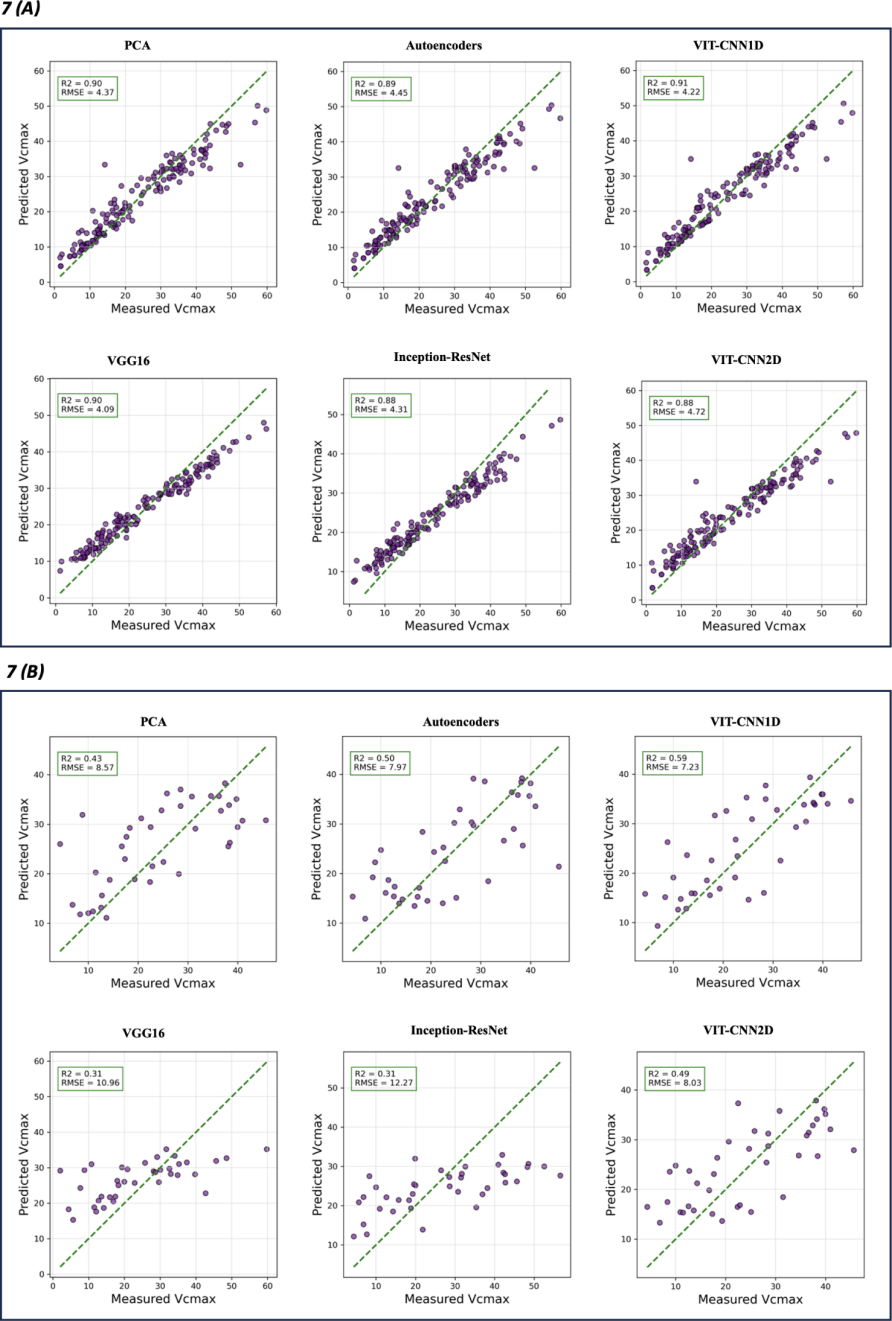
Actual V_cmax_ versus predicted V_cmax_ that were retrieved for different feature extraction algorithms. Training performance **(A)** and test performance **(B)** for each model type. The green dashed line represents the ideal prediction where predicted values perfectly match the measured ones. Evaluation metrics R^2^ and RMSE are on top left of each model graphic.

**Figure 8:**
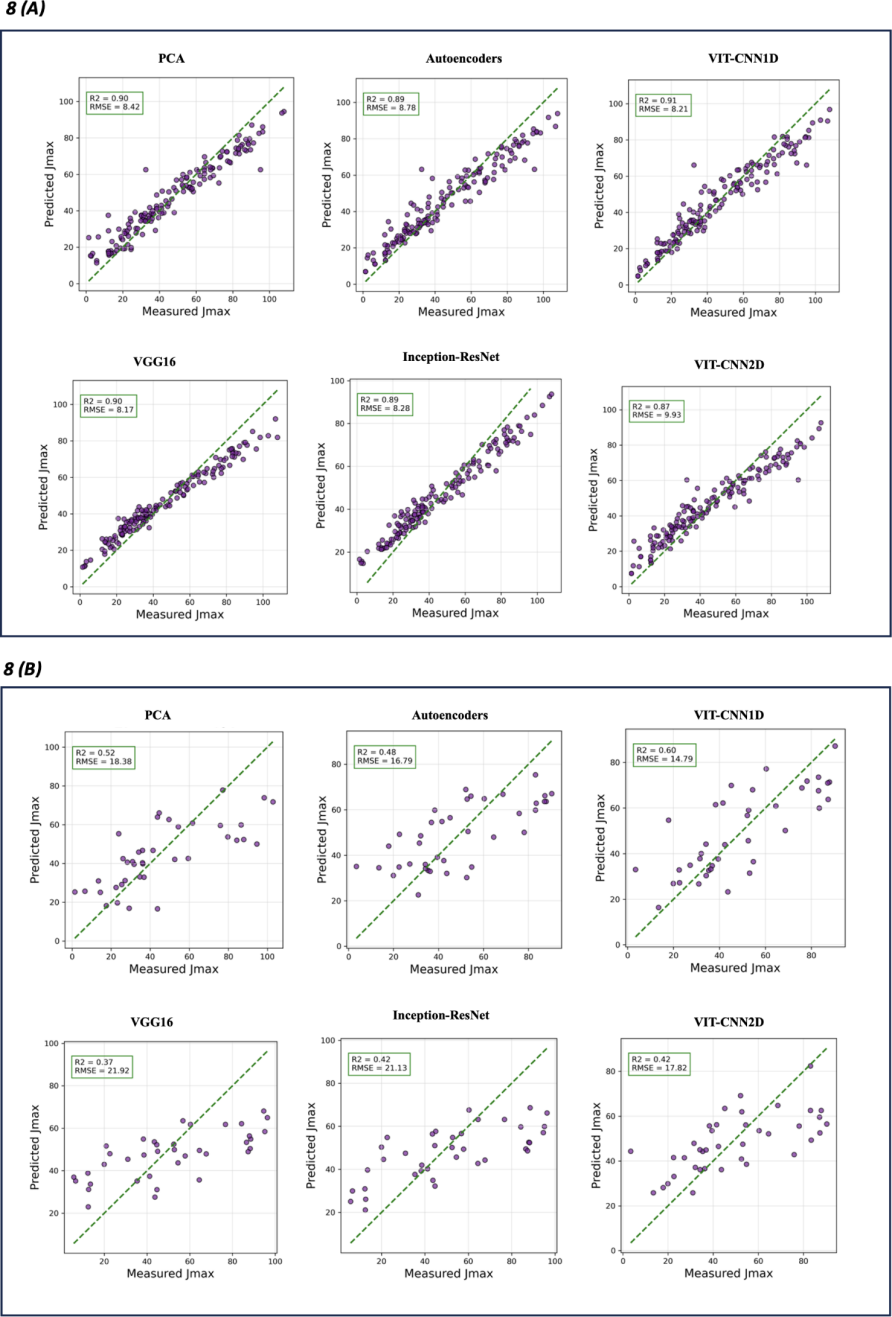
Actual J_max_ versus predicted J_max_ retrieved for each feature extraction algorithms. Training performance **(A)** and test performance **(B)** for each model type. The green dashed line represents the ideal prediction where predicted values perfectly match the measured ones. Evaluation metrics R^2^ and RMSE are on top left of each model graphic.

Of the feature extraction algorithms,VIT-CNN1D model acquired the highest R² value of 0.91 on the training set for predicting V_cmax_ (**Table 4**) This indicated that the model accounted for 91% of the variability in the data. VIT-CNN1D demonstrated the lowest Root Mean Square Error (7.23) and Mean Absolute Error (5.92) on the test set, indicating a robust prediction capability and a high level of generalization to test data. **Figure 7** displays a comparison between the measured V_cmax,_ and the predictions made by various algorithms for both training (**Figure 7A**) and test performance (**Figure 7B**). Likewise, for J_max_ estimation, the VIT-CNN1D model performed best in terms of test RMSE (14.79) and exhibited a competitive test MAE (12.09), reinforcing its robustness across different types of predictions (**Table 4** and **Figure 8**). Considering the test R^2^ among the various algorithms showed that VIT-CNN1D had the highest value for both V_cmax_ (0.59) and J_max_ (0.60) predictions.

**Table 4:** Model performance for training and test dataset used for the prediction of maximum rate of carboxylation of RuBP (V_cmax_) and the maximum rate of electron transport driving RuBP regeneration (J_max_).

V_cmax_ Prediction	Training performance			Test Performance		
Model	R^2^	RMSE	MAE	R^2^	RMSE	MAE
**PCA**	0.89	4.37	3.21	0.43	8.57	6.73
**Autoencoders**	0.89	4.45	3.27	0.50	7.97	5.96
**VIT-CNN1D**	0.91	4.22	3.03	0.59	7.23	5.92
**VGG16**	0.90	4.09	3.09	0.31	10.96	7.32
**InceptionResNet**	0.88	4.31	3.19	0.31	12.27	7.69
**VIT-CNN2D**	0.88	4.72	3.59	0.49	8.03	6.83
J_max_ Prediction	Training performance			Test Performance		
Model	R^2^	RMSE	MAE	R^2^	RMSE	MAE
**PCA**	0.90	8.41	6.41	0.52	18.38	14.67
**Autoencoders**	0.89	8.78	6.68	0.55	15.67	12.77
**VIT-CNN1D**	0.91	8.21	6.13	0.6	14.79	12.01
**VGG16**	0.90	8.17	6.29	0.37	21.92	18.74
**InceptionResNet**	0.89	8.28	6.94	0.42	21.13	17.27
**VIT-CNN2D**	0.87	9.93	7.89	0.42	17.81	14.54

Each of model type mentioned is feature extractor type which was integrated with XGBoost to retrieve model prediction results. The model performance metrics for both training and test dataset are provided.

Comparison of other feature extraction algorithms used for one dimensional hyperspectral data, indicated that PCA showed a strong ability to explain variance in training data but did not perform well in test data in contrast to Autoencoders and VIT-CNN1D. Autoencoders showed some improvement over PCA, but VIT-CNN1D had the lowest RMSE for test performance indicating the best overall predictive capability for both V_cmax_ and J_max_.

The transfer learning algorithms (VGG16 and InceptionResNet) used for feature extractions from images had greater RMSE and MAE for the test set than VIT-CNN1D, PCA, and Autoencoders (**Table 4**). This showed that, while they are effective for image recognition, they may not be the best fit for this specific feature extraction task. Also, VIT-CNN2D model showed improvement over transfer learning approaches but it did not perform as well as the VIT-CNN1D algorithm.

#### 3.3.2 Integration of additional chlorophyll and weather features with the best performing VIT-CNN1D model

**Figure 9:**
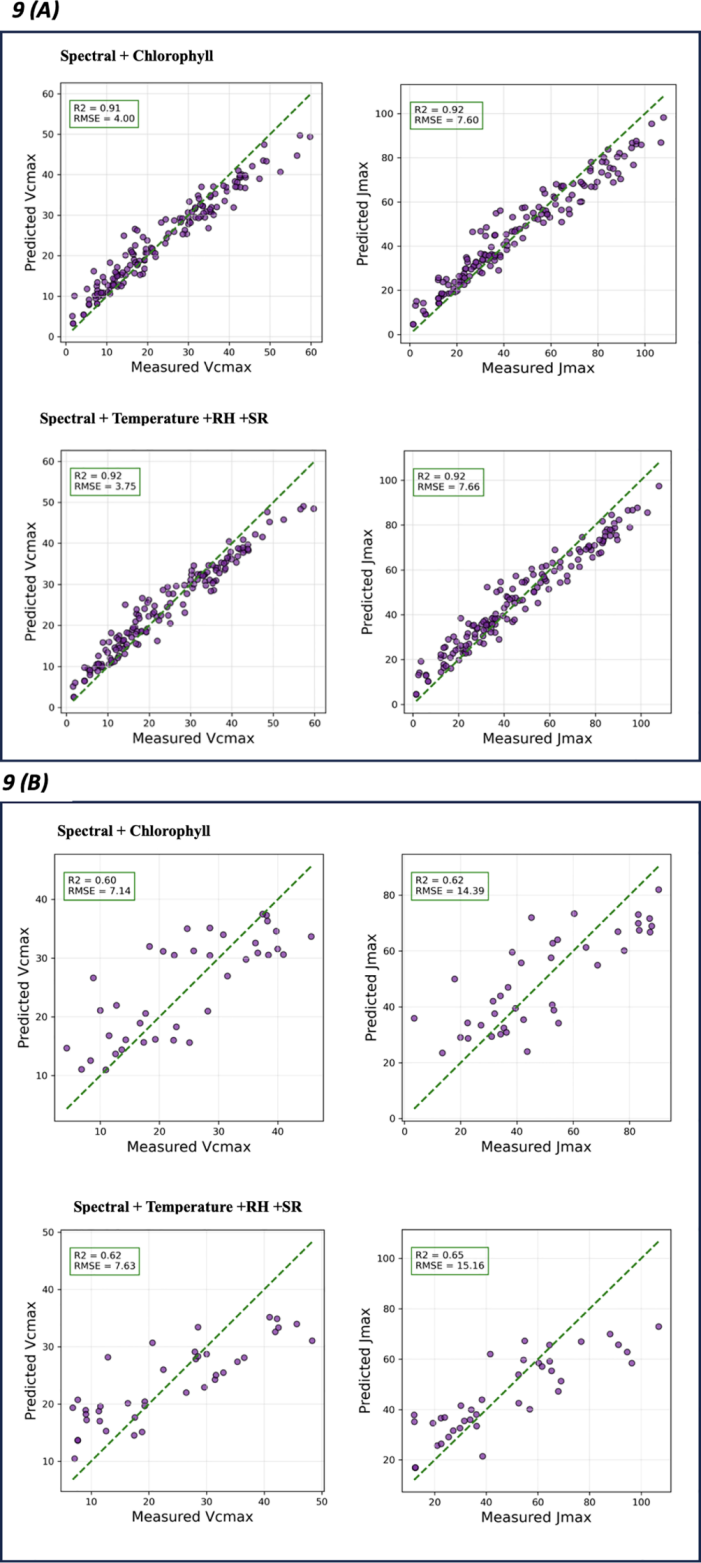
Actual versus predicted plots retrieved for V_cmax_ and J_max_ using VIT-CNN1D + XGBoost. Training performance **(A)** and test performance **(B)** for each model type. The green dashed line represents the ideal prediction where predicted values perfectly match the measured ones. Evaluation metrics R^2^ and RMSE are on top left of each model graphic.

The ground-truth photosynthetic parameter and hyperspectral data were collected across the growing season which included a gradually decreasing daylength, temperature, SR, and variable RH. Chlorophyll concentration was relatively similar in concentration across the ‘Marquette’_rootstock combinations (**Supplementary Figure 1A**), although it appeared to decrease at 12h daylength (**Supplementary Figure 1B**). Solar radiation (SR) was lower in 2023 than 2022 at 12h daylength and RH was greater at the 12 h daylength in 2023 than in 2022 (**Supplementary Figures 3, 4**). Addition of these parameters into the best performing model VIT-CNN1D resulted in small changes in model performance (**Figure 9**). The V_cmax_ prediction increased the training and test performance slightly with R^2^ values of 0.91 to 0.92 and 0.60 to 0.62, respectively across the spectral + chlorophyll, and spectral + temperature + RH + SR feature sets (**Table 5**). The test RMSE and MAE for V_cmax_ were comparable or lower with the addition of chlorophyll and slightly higher with the addition of weather parameters. The performance of VIT-CNN1D with consideration of chlorophyll and weather parameters for J_max_ exhibits a comparable pattern. The training R^2^ values for model are similar for both the spectral and spectral plus chlorophyll and spectral plus weather feature sets (**Table 5**). Test performance however showed an increased R^2^ with variable RMSE and MAE that were greater or less than the spectral input alone. The variability of RMSE and MAE in training and testing for these additional features suggested the possibility of overfitting and indicated the importance of refining the model or collecting more data to enhance its ability to make accurate predictions for both V_cmax_ and J_max_.

**Table 5:** Model performance for training and test dataset used for the prediction of maximum rate of carboxylation of RuBP (V_cmax_) and the maximum rate of electron transport driving RuBP regeneration (J_max_).

Prediction Model	Training performance			Test Performance		
	R^2^	RMSE	MAE	R^2^	RMSE	MAE
**V_cmax_ Spectral + Chlorophyll**	0.91	4.00	3.02	0.60	7.14	6.08
**V_cmax_ Spectral + Temperature + RH + SR**	0.92	3.75	3.03	0.62	7.62	6.40
**J_max_ Spectral + Chlorophyll**	0.92	7.59	5.86	0.62	14.39	12.25
**J_max_ Spectral + Temperature + RH + SR**	0.91	7.66	6.26	0.65	15.15	11.66

Each set of results represents differences in model input features: Spectral + Chlorophyll and Spectral + Temperature + RH + SR and the training and test performance metrics.

## 4 Discussion

‘Marquette’_3309C and ‘Marquette’_1103P had the highest average value for both V_cmax_ and J_max_, while ‘Marquette’_5C and ‘Marquette’_SO4 had the lowest average value and indicated differences in photosynthetic performance. The results from *post-hoc* analysis was particularly meaningful and indicated differences in rootstock genotype influence on scion photosynthesis. The rootstock genotypes displayed different levels of photosynthetic efficiency representing a conferred rootstock impact on the structure and function of the photosynthetic machinery in the scion. In accordance with these findings, when comparing other physiological measures like stomatal conductance to water vapor and net assimilation rate among these genotypes in the same settings, there was significant difference between their performances. It was observed in another study, that Marquette on 3309C and 1103P exhibited the highest net assimilation rate and stomatal conductance, which are direct measurements of the physiological processes associated with photosynthesis (Sharma et al., 2024). Many studies of grapevine rootstock’s influence on photosynthesis are based on stress treatment conditions in comparison to an unstressed control (da Silva et al., 2018; Prinsi et al., 2021). Rootstocks are found to alter the plant’s response to physiological stress conditions which possibly leads to reduction of photosynthetic efficiency through stomatal and non-stomatal limitations (Dinis et al., 2018; Tombesi et al., 2019). In this study with no intentional stress treatments, the rootstock genotypes conferred significant differences in scion’s photosynthetic efficiency. This implied that there is a genetic basis underlying rootstock influence on photosynthetic parameters. Similarly, Pou et al. (2022) show in *V. vinifera* ‘Tempranillo’ 30-year-old vines, on four different rootstocks, that gas exchange parameters, vine vigor, and chlorophyll content is influenced by rootstock genotype. Like these findings, our results suggested that the rootstocks with a *V. rupestris X V. riparia* pedigree (1103P and 3309C, **Table 1**) had significantly increased photosynthetic performance over those with a *V. berlanderi x V. riparia* pedigree (5C and SO4). In this study, it is not possible to determine whether it is *V. rupestris, V. berlanderi, V. riparia*, or an interaction of the different species in the rootstocks genetic makeup that resulted in the conferred photosynthetic difference. Thus, it is crucial to conduct comprehensive, locale-specific, long-term research due to the complex chain of interactions among the rootstock, the scion cultivar, and the environmental conditions before recommending a specific rootstock for a given set of soil and climatic conditions.

The PCA results demonstrated the effectiveness of hyperspectral imaging in distinguishing genotypes according to their photosynthetic efficacy (**Figure 6**). Through distinct clustering, the PCA score plots for different years highlighted the variability in photosynthetic responses across genotypes and environmental conditions. The detailed spectral information and subtle differences in their signature patterns obtained through hyperspectral imaging helped to differentiate genotypes. Despite extensive research, the precise physiological signals captured by hyperspectral reflectance for predicting V_cmax_ remain poorly understood (Meacham-Hensold et al., 2020). So far, Predictive models developed have consistently identified significant wavelengths in the visible (400–700 nm) and red-edge (700–740 nm) regions, areas typically associated with pigment content (Barnes et al., 2017; Ely et al., 2019; Meacham-Hensold et al., 2019). Similar to these finding, the loadings plot (**Figure 6B**) offered an insight into the specific wavelengths that were most indicative of these differences; the peaks corresponding to the spectral features of chlorophyll (approximately 710 nm) and carotenoids (approximately 460 nm) (Falcioni et al., 2023) as well as the NIR region at 760 nm, which is indicative of other physiological properties related to photosynthetic efficiency (Sexton et al., 2021). The changes in PCA plots from 2022 to 2023 showed how hyperspectral imaging can record changes in photosynthetic efficiency over time. These changes could be caused by environmental factors or changes in the scions’ development.

Research related to validation of hyperspectral data as indirect measure of photosynthesis is very limited in grapevines (Yang et al., 2022). Validation analysis of indirect measures of photosynthesis in this study indicated that there was a relationship between hyperspectral data and direct measured photosynthetic parameters. The best performing model VIT-CNN1D was able to explain around ~60% variation in test dataset for both parameters. In contrast, estimation of V_cmax_ and J_max_ for *Populus* species using leaf-level hyperspectral data show that the best model had R^2^ value of 0.51 and 0.54, respectively (Kyaw et al., 2022). Meacham-Hensold et al. (2020) show that the hyperspectral data retrieved from sunlit section of *Nicotiana tabacum*, yielded an R^2^ 0.79 for V_cmax_ and an R^2^ of 0.59 for J_max_ using partial least square regression model. However, the *Nicotiana tabacum* study used transgenic lines with genetically altered photosynthetic pathways, thus were able to capture greater photosynthetic variability, in contrast to our common scion grafted grapevine research. Developing trait values with intentionally modified photosynthetic qualities in an ungrafted plant offers the models a potentially more simplified prediction goal. In this study, the vines although fruiting were relatively young and might still be undergoing changes, as the vine structure continues to mature in their site, that may affect the influence of its rootstock on photosynthesis. However, it is noted that vineyards with 30-year-old ‘Tempranillo’ vines grafted on 1103P show higher net photosynthetic rate than other less vigorous rootstocks (Pou et al., 2022). Similarly, we show a greater V_cmax_ and J_max_ in ‘Marquette’ on 1103P than ‘Marquette’ on 5C, SO4 or Freedom rootstocks. Further studies through time will be needed to determine if differences identified will remain through the life a vine and to increase confidence of the use of hyperspectral imagery to measure photosynthesis in long- lived perennial plants in diverse geographical locations.

A comparison of the PCA, autoencoders and VIT-CNN1D algorithms, all having one-dimensional hyperspectral data as input indicated that VIT-CNN1D had the best model performance in both training and test dataset as compared to PCA and autoencoders. The working approach of a 1D-CNN includes a convolutional filter (or kernel) moving across the one-dimensional input data where at each position it executes a multiplication of elements and then combines the results into a single output value. As the convolutional filters move across the input, they can extract important features (such as specific patterns) from the data. In our analysis, the VIT-CNN1D architecture, with the potential to detect sequential patterns in the hyperspectral data, gave it an advantage over PCA and autoencoders which do not account for the inherent order of data points. Deep learning models like CNN are shown to have better prediction accuracies as compared to traditional machine learning approaches (Kumar et al., 2020). 1D-CNNs can learn a hierarchy of features, in contrast to PCA, which takes a linear approach, and Autoencoders, which are usually shallow in comparison to deep CNNs. This implies that 1D-CNNs are able to identify intricate patterns across various scales, identifying both local and global characteristics in sequential data (Kiranyaz et al., 2021). Likewise, VIT-CNN1D outperformed other transfer learning models and the VIT-CNN2D algorithm, used for image-based feature extraction, in this study. Two-dimensional deep convolutional neural networks (2D-CNNs) are specifically effective in addressing computer-vision issues, directly using the raw image as input without any manual preprocessing. The convolutional layer in 2D-CNN performs feature extraction through a combination of several linear and nonlinear algorithms applied using activation function. To spatially compress the input volume, the pooling layers determine the maximum (max pooling) or average (average pooling) value in the neighborhood pixels. This helps to decrease the dimensionality of the maps, thus decreasing the complexity of their computation. However, in order to achieve robust performance for 2D-CNN requires an extensive number of training samples (Alzubaidi et al., 2020, 2021). One of the our study’s limitations lies in the lower number of ground-truth samples, as biological data for direct physiological measures are very time-consuming to acquire and hence difficult to gather in a given timeframe (Haworth et al., 2023). VIT-CNN1D had fewer parameters compared to other 2D-CNN based algorithms making it more efficient with limited training samples (Kiranyaz et al., 2019; Wang et al., 2017). An additional reason for superior performance of VIT-CNN1D over VIT-CNN2D could be that the photosynthetic measures in this case are more correlated to the spectral features rather than the spatial features.

In this study, the ground-truth photosynthetic parameter and hyperspectral data were collected across the growing season; therefore, it was important to consider chlorophyll concentration and environmental factors. The model metrics and additional parameters (chlorophyll concentration or temperature, HR, and SR), did not drastically alter the model’s performance. Many studies use chlorophyll content as proxy for photosynthetic activity parameter (Mandal and Dutta, 2020; Prilianti et al., 2021; Shi et al., 2022). The test R^2^ prediction increased slightly for V_cmax_ and J_max_ with a decrease in RMSE with the addition of chlorophyll. The addition of weather parameters to the VIT-CNN1D model provided more mixed results with the test R^2^, RMSE and MAE varying little for V_cmax_ and J_max._ Several studies (Bassow and Bazzaz, 1998; Serbin et al., 2012), show that “biophysical” parameters measured at leaf level are directly associated with photosynthesis. The emphasis of our research was on broad physical parameters associated with weather conditions. Although these parameters are noteworthy, they do not cover the entire range of factors that impact the phenomena of photosynthesis. To attain greater comprehension, prospective research should attempt to integrate measurements of leaf-level biophysical parameters. These factors encompass internal leaf structure, stomatal density, and leaf temperature, as well as incoming photosynthetically active radiation, among others.

## 5 Conclusions

The effect of rootstock mediation on the photosynthesis of scion is an important topic in viticulture due to the opportunity it offers for selection and identification of rootstocks that can improve scion response to a changing climate. This study investigated common scion photosynthetic measures with different rootstock combinations, leveraging two different methodological approaches: direct measures through IRGA and indirect measures using hyperspectral remote sensing. The study also examined the efficacy of indirect measurements in different environments and verified its validity through the integration of multiple AI/computer vision algorithms. Comprehensively, the following are significant findings derived from this research: 1. Across two growing seasons, substantial variation in photosynthetic efficacy (V_cmax_ and J_max_) was observed for six distinct ‘Marquette’_rootstock combinations across four daylengths. This suggested that the rootstock genotype exhibits a significant influence on the scion physiological response related to photosynthesis. Similarly, substantial variation in hyperspectral signature was observed among graft combinations relative to the rootstock genotypes. Both direct and indirect measures were hugely influenced by daylength conditions in all graft combinations. 2. To derive spectral and spatial features, numerous feature extraction algorithms were evaluated and VIT-CNN1D demonstrated the greatest potential with an R^2^ of 0.60 for both parameters. Spatial feature extraction models, namely VGG16, Inception-ResNet, and VIT-CNN2D, exhibited subpar performance due to their restricted training samples and the lack of association between response variables and spatial relationships. 3. Incorporating additional input features of chlorophyll gave a small improvement in training and test performance in contrast to the weather parameters. This study highlights the substantial impact of rootstock genotype on the photosynthetic efficacy of scion plants, indicating that the selection of suitable rootstocks can improve the resilience of vineyards to climate change. By utilizing AI algorithms to validate hyperspectral remote sensing, the research reflects the potential for nondestructive, efficient monitoring techniques in viticulture. The substantial influence of daylength on photosynthetic measures shows the necessity of considering whole growing season environmental factors when selecting rootstocks. Future recommendations include the following: conducting thorough rootstock trials, optimizing hyperspectral imaging and AI models, integrating environmental data, and leveraging chlorophyll and pigment data to continuously improve the nondestructive assessment of photosynthesis.

## Data Availability

The raw data supporting the conclusions of this article will be made available by the authors, without undue reservation.
