# Supplementary material for: Hyperspectral imaging and artificial intelligence enhance remote phenotyping of grapevine rootstock influence on whole vine photosynthesis
Source: Front Plant Sci. 2024 Sep 19;15:1409821. doi: 10.3389/fpls.2024.1409821 (PMC11446806; doi:10.3389/fpls.2024.1409821)
Supplement: Supplementary file 1 [file Image1.pdf]

Supplementary Material

1 Supplementary Figures

Supplementary Figure 1A.

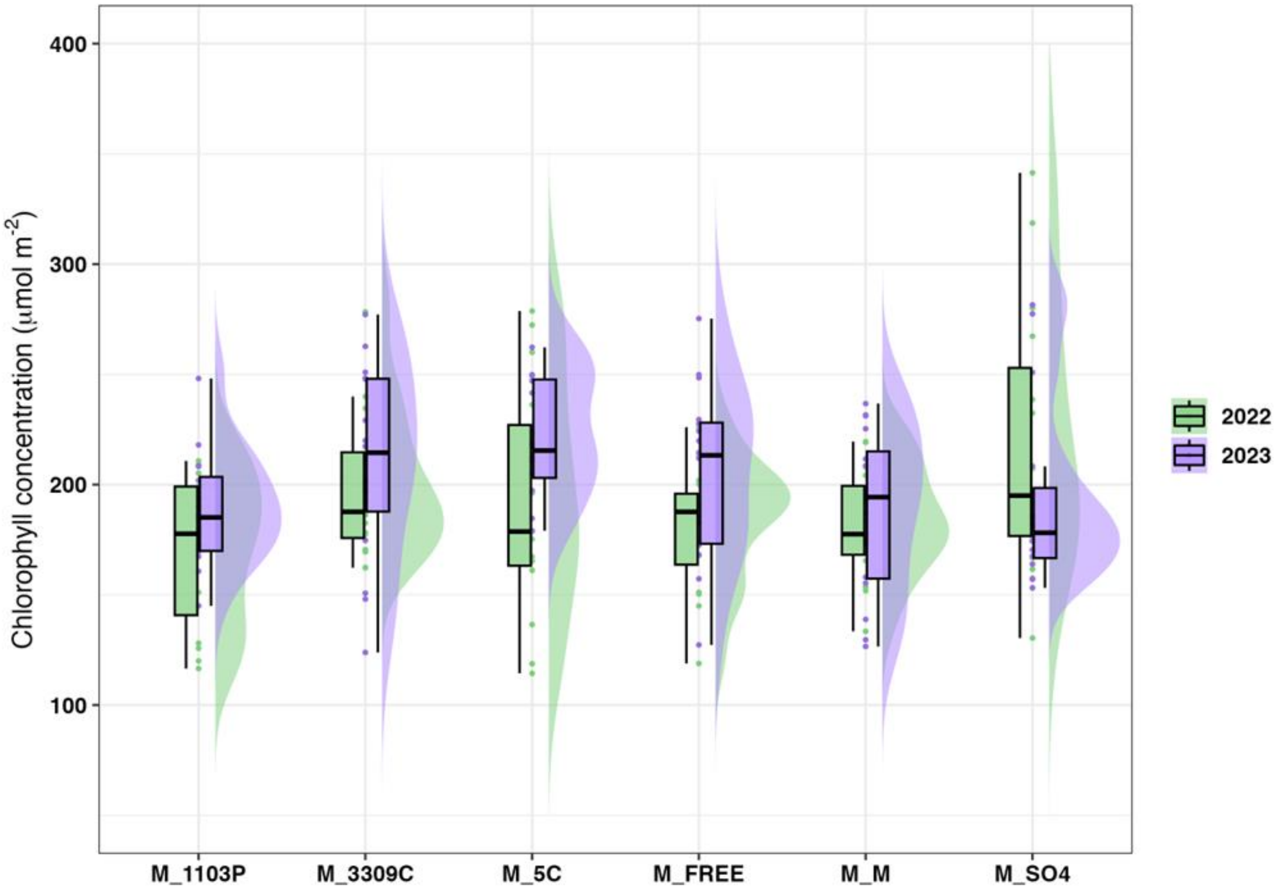

Supplementary Figure 1B

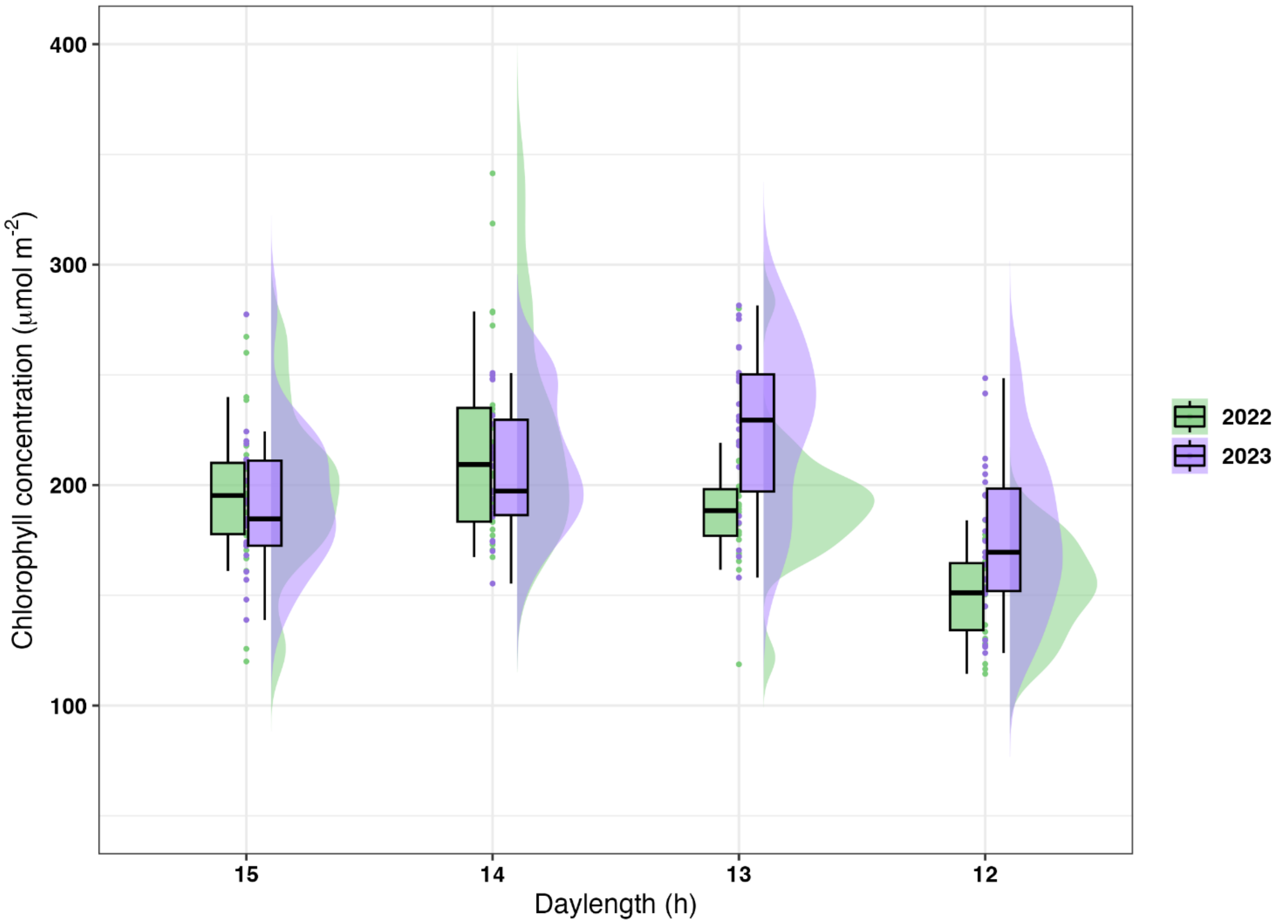

Supplementary Figure 1A and B. Distribution of chlorophyll concentration (CC) across rootstock combinations and daylengths. A) all graft combinations, *V. hybrid* 'Marquette' heterografted to rootstock 1103P, 3309C, 5C, Freedom (FREE), and SO4 and homografted to 'Marquette' across all daylengths, n=4; B) Temporal trend of CC across all six graft combinations (n=4) for each daylength; year 2022 (green), 2023 (purple).

Supplementary Figure 2.

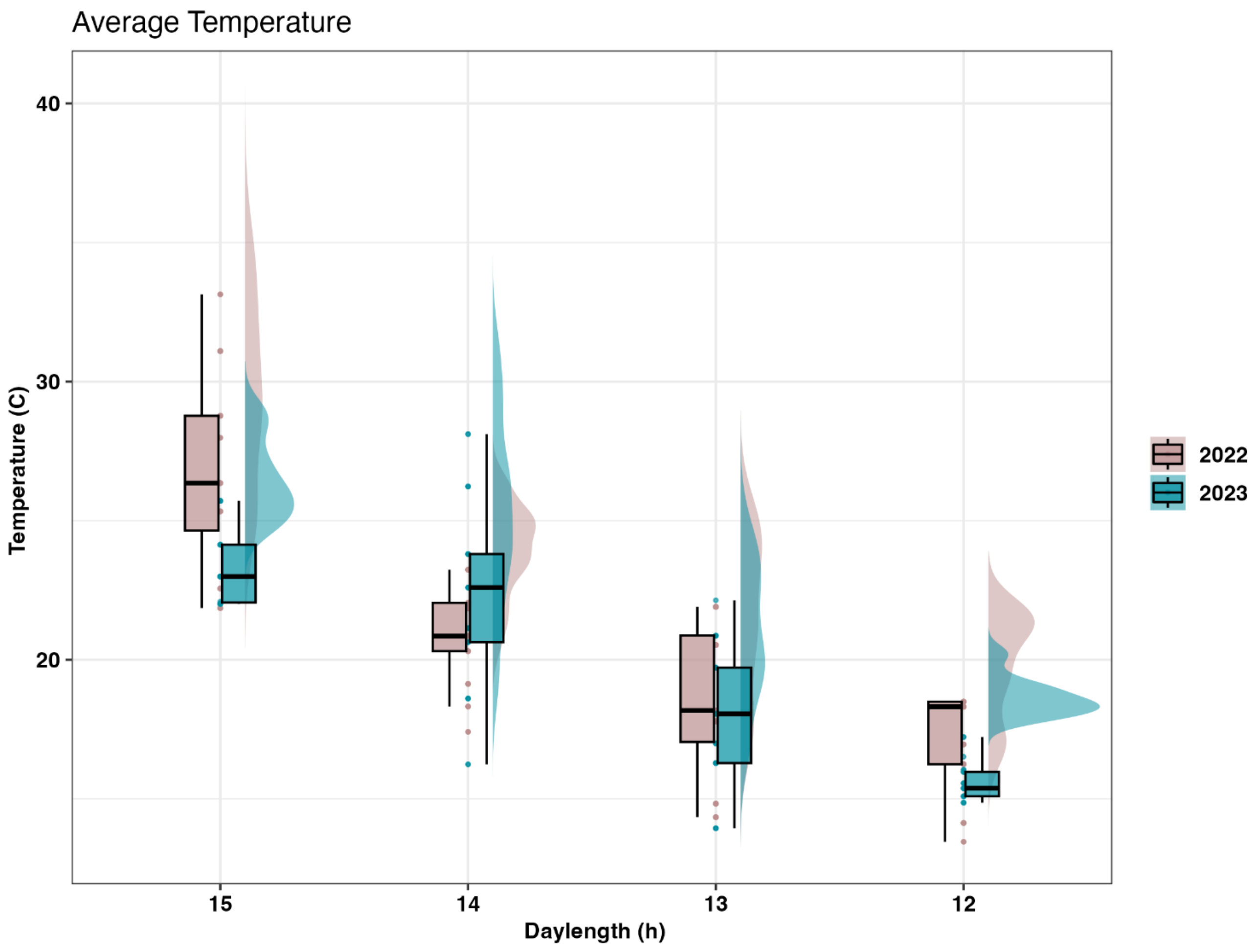

Supplementary Figure 2. Temporal trend in average temperature at each of the photosynthetic measurements. Each daylength includes the average temperature for all six graft combinations and their replicates; year 2022 (pink) and 2023 (blue).

Supplementary Figure 3.

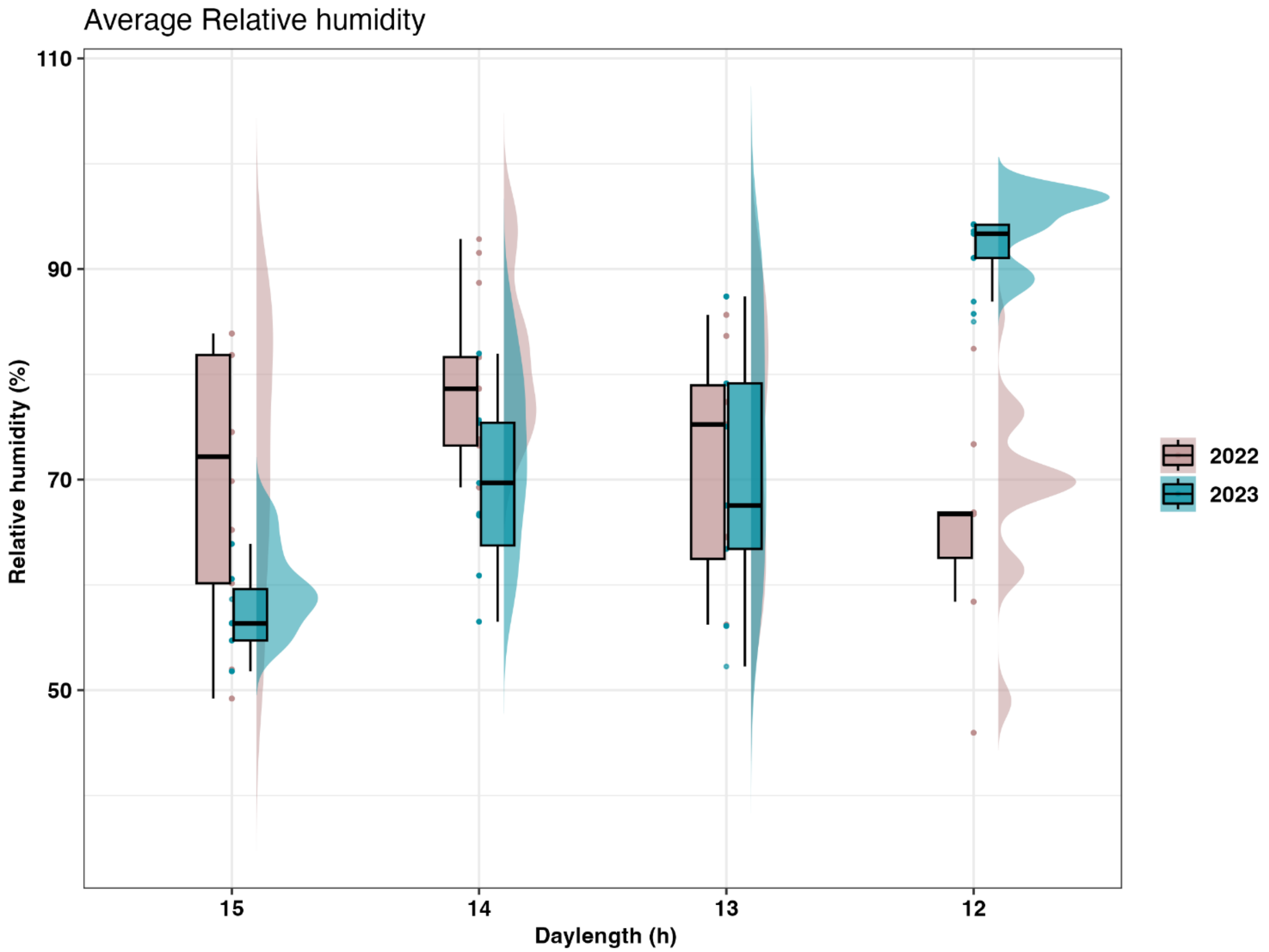

Supplementary Figure 3. Temporal trend in average relative humidity (RH) at each of the photosynthetic measurements. Each daylength includes the average temperature for all six graft combinations and their replicates; year 2022 (pink) and 2023 (blue).

Supplementary Figure 4.

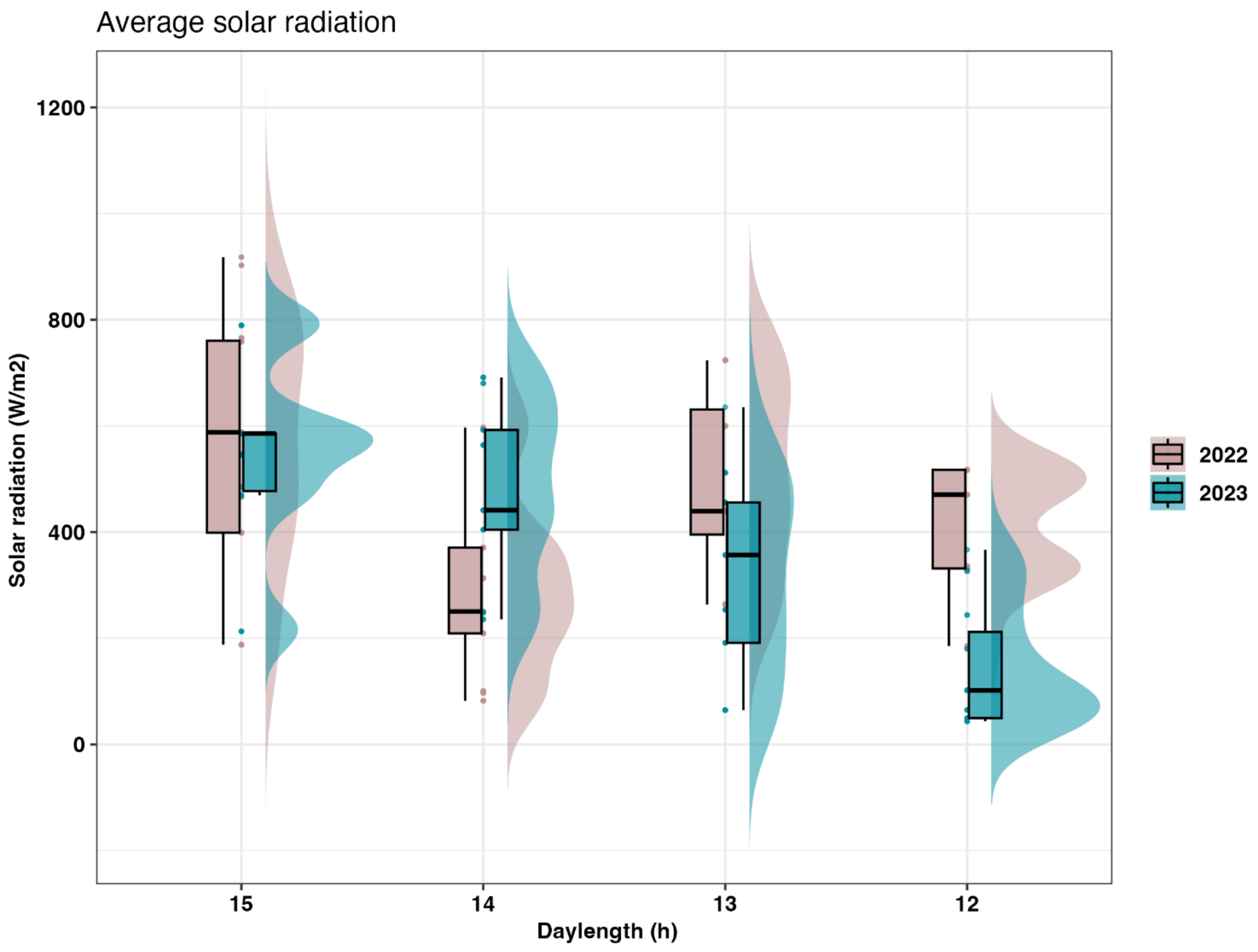

Supplementary Figure 4. Temporal trend for average solar radiation (SR) at each of the photosynthetic measurements. Each daylength includes the average temperature for all six graft combinations and their replicates; year 2022 (pink) and 2023 (blue).
